# Dissection of a Quantitative Trait Locus for PR Interval Duration Identifies *Tnni3k* as a Novel Modulator of Cardiac Conduction

**DOI:** 10.1371/journal.pgen.1003113

**Published:** 2012-12-06

**Authors:** Elisabeth M. Lodder, Brendon P. Scicluna, Annalisa Milano, Albert Y. Sun, Hao Tang, Carol Ann Remme, Perry D. Moerland, Michael W. T. Tanck, Geoffrey S. Pitt, Douglas A. Marchuk, Connie R. Bezzina

**Affiliations:** 1 Heart Failure Research Center, Department of Experimental Cardiology, Academic Medical Center, University of Amsterdam, Amsterdam, The Netherlands; 2 The Ion Channel Research Unit, Department of Medicine, Duke University, Durham, North Carolina, United States of America; 3 Department of Molecular Genetics and Microbiology, Duke University, Durham, North Carolina, United States of America; 4 Department of Clinical Epidemiology, Biostatistics, and Bioinformatics, Academic Medical Center, University of Amsterdam, Amsterdam, The Netherlands; Stanford University School of Medicine, United States of America

## Abstract

Atrio-ventricular conduction disease is a common feature in Mendelian rhythm disorders associated with sudden cardiac death and is characterized by prolongation of the PR interval on the surface electrocardiogram (ECG). Prolongation of the PR interval is also a strong predictor of atrial fibrillation, the most prevalent sustained cardiac arrhythmia. Despite the significant genetic component in PR duration variability, the genes regulating PR interval duration remain largely elusive. We here aimed to dissect the quantitative trait locus (QTL) for PR interval duration that we previously mapped in murine F2 progeny of a sensitized 129P2 and FVBN/J cross. To determine the underlying gene responsible for this QTL, genome-wide transcriptional profiling was carried out on myocardial tissue from 109 F2 mice. Expression QTLs (eQTLs) were mapped and the PR interval QTL was inspected for the co-incidence of eQTLs. We further determined the correlation of each of these transcripts to the PR interval. *Tnni3k* was the only eQTL, mapping to the PR-QTL, with an established abundant cardiac-specific expression pattern and a significant correlation to PR interval duration. Genotype inspection in various inbred mouse strains revealed the presence of at least three independent haplotypes at the *Tnni3k* locus. Measurement of PR interval duration and *Tnni3k* mRNA expression levels in six inbred lines identified a positive correlation between the level of *Tnni3k* mRNA and PR interval duration. Furthermore, in DBA/2J mice overexpressing h*TNNI3K*, and in DBA.AKR.*hrtfm2* congenic mice, which harbor the AKR/J “high-*Tnni3k* expression” haplotype in the DBA/2J genetic background, PR interval duration was prolonged as compared to DBA/2J wild-type mice (“low-*Tnni3k* expression” haplotype). Our data provide the first evidence for a role of *Tnni3k* in controlling the electrocardiographic PR interval indicating a function of *Tnni3k* in atrio-ventricular conduction.

## Author Summary

Atrio-ventricular (AV) conduction disease (delay), characterized by prolongation of the PR interval on the surface electrocardiogram (ECG), is a common feature in Mendelian rhythm disorders and is associated with sudden cardiac death. Prolongation of the PR interval is also a strong predictor of atrial fibrillation (AF), the most common sustained cardiac arrhythmia. Although there is a substantial heritable component to the variability of the PR interval, the causative genes remain largely elusive. The identification of these genetic factors in the human population has been difficult owing to wide genetic heterogeneity and an uncontainable environment. We here exploited the homogeneous genetic background and controlled environment of inbred laboratory mouse strains to detect a genetic modifier of the PR interval. We identify *Tnni3k* as prime candidate for the modulation of the PR interval duration and suggest a new role for this gene, in the modulation of atrio-ventricular conduction.

## Introduction

Atrio-ventricular (AV) conduction delay describes the impairment of the electrical continuity between the atria and the ventricles and is characterized by prolongation of the PR interval on the surface electrocardiogram (ECG). AV delay of varying severity is a common feature in Mendelian rhythm disorders and is associated with sudden cardiac death [1]. PR interval prolongation is also a strong predictor of atrial fibrillation (AF) [2] and is therefore considered an intermediate phenotype for this condition [3]. AF is the most commonly observed sustained cardiac arrhythmia, with an age dependent prevalence of up to 9% [4]. Identification of genetic determinants of AV conduction delay is essential for understanding the underlying molecular mechanisms and for the possibility of development of targeted treatments and prevention strategies.

There is a strong heritable component in the variability of the PR interval [5]–[7] and although genome-wide approaches have highlighted several causal loci [3], a major proportion of the heritability and the underlying genes remains elusive. The identification of these genetic factors in the human population has been difficult owing to wide genetic heterogeneity and an uncontainable environment. We here exploit the homogeneous genetic background and controlled environment of inbred laboratory mouse strains to identify a novel genetic modifier of the PR interval.

We have previously detected a quantitative trait locus (QTL) for the PR interval (PR-QTL) on chromosome 3 in a conduction disease sensitized mouse F2 progeny of mice harboring the cardiac voltage-gated sodium channel gene mutation *Scn5a* ^1798insD/+^, generated from a 129P2-*Scn5a* ^1798insD/+^×FVBN/J-*Scn5a* ^1798insD/+^ cross [8]. 129P2-*Scn5a* ^1798insD/+^ and FVBN/J-*Scn5a* ^1798insD/+^ mice recapitulate many of the electrocardiographic (ECG) manifestations seen in patients carrying the homologous mutation *SCN5A*-1795insD, including cardiac conduction defects. Importantly, 129P2-*Scn5a* ^1798insD/+^ and FVBN/J-*Scn5a* ^1798insD/+^ mice display different severity of conduction disease [9], [10]. These differences in conduction disease severity are likely due to a complex interplay of multiple modifier loci. We previously exploited these strain effects on cardiac conduction to map a QTL on mouse chromosome 3 that influences the variance in PR interval [8]. The aim of the present study was to dissect this QTL in detail to identify the underlying gene responsible for the variation in PR interval.

As genetic variation underlying a QTL may act through effects on gene expression [11], we tested the presence of such variability in our F2 population. We integrated genome-wide transcriptional profiles of myocardial tissue with single nucleotide polymorphism (SNP) mapping in our hybrid mouse population to delineate genetic loci in association with variance in gene expression. These expression QTLs (eQTLs) where assessed for overlap with the PR interval QTL. Of the thus found 16 eQTLs only *Tnni3k* expression levels both correlated to the PR interval and had a high cardiac specific expression.

We integrated genome-wide transcriptional profiles of myocardial tissue with genotypic data in F2 progeny from the 129P2-*Scn5a* ^1798insD/+^×FVBN/J-*Scn5a* ^1798insD/+^ cross to uncover eQTLs that overlapped with the chromosome 3 PR interval QTL. Of these eQTLs, only the transcript for *Tnni3k* both correlated to the PR interval and was highly and specifically expressed in heart; *Tnni3k* was thus identified as a very strong candidate for the effect. The role of *Tnni3k* was subsequently validated *in silico* using phenotypic data from the mouse phenome database [12]. Further validation was performed by testing the correlation of *Tnni3k* expression level with PR interval in 6 inbred mouse strains harboring 3 independent haplotypes at the *Tnni3k* genomic locus. Finally, the role of *Tnni3k* in modulation of the PR interval was validated *in vivo* in (i) congenic mice harboring the high-*Tnni3k* expression haplotype of the AKR/J strain in the DBA/2J (low expression of *Tnni3k*) genetic background and (ii) in DBA/2J mice overexpressing human *TNNI3K.*

## Results

### F2 screen for identifying genetic modifiers of cardiac conduction

The identification of a main-effect PR interval QTL in the distal portion of chromosome 3 has been reported previously [8]. In brief, we combined ECG and genome-wide genotypic data in 502 F2 progeny generated from an FVBN/J-*Scn5a* ^1798insD+/−^×129P2-*Scn5a* ^1798insD+/−^ intercross to map QTLs for ECG parameters. The most significant SNP association with PR interval was rs13477506; explaining 3.9% (*p* = 7.71×10^−5^) of the observed variance in PR interval [8].

### Identification of eQTLs for the PR-QTL on chromosome 3

As genetic variation underlying a QTL may act on the trait through effects on gene expression [11] we tested the presence of such variability in the F2 mice. We measured genome-wide gene expression profiles of cardiac tissue in 109 previously genotyped F2 mice using microarrays. Normalized *log*-transformed intensities of individual transcripts were used as quantitative inputs for eQTL mapping. Considering a multiple-comparison corrected significance threshold of *p*<1.88×10^−6^ (LOD>6.83), we uncovered 16 eQTLs within the 1.5 LOD drop for the PR interval chromosome 3 QTL (Table 1). Of these 16 eQTLs, seven were deemed cis-regulated (cognate gene and eQTL SNP physically map to the same genomic region) indicating a possible direct effect of the underlying genetic variation on transcript levels; whereas nine eQTLs were labeled as trans-regulated (cognate gene physically maps to a different genomic region than the eQTL SNP) suggesting indirect effects such as transcription factor levels influencing the transcript level.

**Table 1:** eQTLs overlapping the 1.5 LOD drop region of the PR QTL.

Gene symbol	Refseq ID	Gene name	Max LOD	SNP marker	eQTL cis/trans	Spearmans Corr. to PR rho	pvalue
***Eif4e***	**NM_007917**	**eukaryotic translation initiation factor 4E**	**21.58**	**rs6407142**	**cis**	**−0.246**	**0.01**
*Gbp1*	NM_010259	guanylate binding protein 1	9.3	rs6407142	cis	0.128	0.19
*Ccbl2*	NM_173763	cysteine conjugate-beta lyase 2	19.77	rs13477494	cis	−0.001	0.99
*Cyr61*	NM_010516	cysteine rich protein 61	7.54	rs13477494	cis	−0.076	0.43
*Myoz2*	NM_021503	myozenin 2	12.1	rs13477494	trans	−0.104	0.28
***Gipc2***	**NM_016867**	**GIPC PDZ domain containing family, member 2**	**8.05**	**rs13477494**	**cis**	**−0.300**	**0.00**
*Abca4*	NM_007378	ATP-binding cassette, sub-family A (ABC1), member 4	12.63	rs13477494	trans	0.105	0.28
*Shmt1*	NM_009171	serine hydroxymethyltransferase 1 (soluble)	6.94	rs13477506	trans	0.093	0.33
*Bcl6*	NM_009744	B-cell leukemia/lymphoma 6	9.71	rs13477506	trans	−0.218	0.02
***Socs2***	**NM_001168655**	**suppressor of cytokine signaling 2**	**25.31**	**rs13477506**	**trans**	**0.279**	**0.00**
*Igfals*	NM_008340	insulin-like growth factor binding protein, acid labile subunit	21.43	rs13477506	trans	0.184	0.06
*Esrrb*	NM_001159500	estrogen related receptor, beta	7.17	rs13477506	trans	0.102	0.29
*3110057O12RIK*	NM_026622	RIKEN cDNA 3110057O12 gene	10.34	rs13477506	trans	0.233	0.02
***Tnni3k***	**NM_177066**	**TNNI3 interacting kinase**	**56.46**	**rs13477506**	**cis**	**0.263**	**0.01**
*Extl2*	NM_001163514	exostoses (multiple)-like 2	8.01	rs13477506	trans	0.044	0.65
*Gpr177*	NM_026582	G protein-coupled receptor 177	7.93	CEL.3_159340478	cis	−0.175	0.07

Transcripts with a significant linkage overlapping the 1.5 LOD drop region of the PR QTL are listed with their genomic location (build NCBIM37), specific LOD score, marker with the highest LOD score, Spearmans correlation to PR (Rho and uncorrected p value). Transcripts with a significant correlation are marked in bold; *Tnni3k* is marked in bold and underlined.

To determine whether any of the identified eQTLs could be a candidate for the effect of the Chr3 PR-QTL we tested correlation of the transcript levels with the PR interval duration. Only four transcripts showed significant correlation to the PR interval, namely, *Eif4e* (rho −0.246, p<0.01), *Gipc2* (rho −0.300, p<0.001), *Socs2* (rho 0.279, p<0.001) and *Tnni3k* (rho 0.263, p<0.01). Data from the GEO database indicates that of these only *Tnni3k* displays a high cardiac specific expression pattern [13], [14]. We therefore investigated *Tnni3k* further as the prime candidate for the effect at the chromosome 3 PR-QTL.

**Figure 1:**
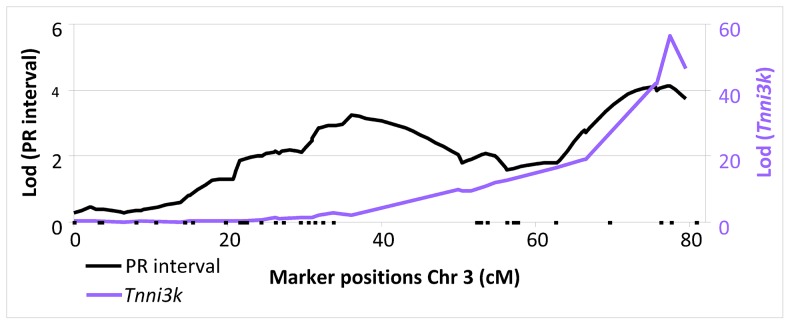
LOD plot of PR interval (black, left y-axis) and *Tnni3k (purple* right y-axis) on chromosome 3.

**Figure 2:**
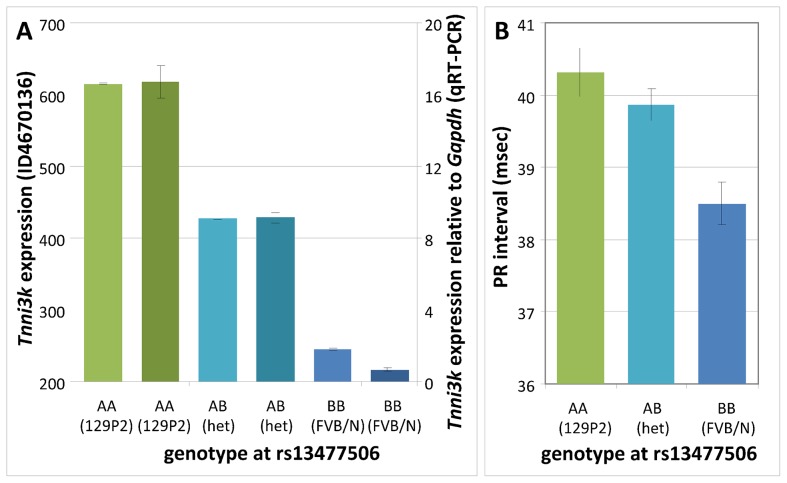
Genotype effects at rs13477506. (A) *Tnni3k* expression (Illumina probe ILMN_3023962) as a function of genotype at rs13477506 in F2 mice; homozygous 129P2: AA, green; 129P2-FVBN/J heterozygous: AB, turquoise; homozygous FVBN/J: BB, blue; the darker shades represent the independent validation of the *Tnni3k* transcription levels by Q-PCR (right y-axis). (B) PR interval as a function of genotype at rs13477506 in F2 mice. Error bars indicate standard errors.

Inspection of the genome-wide LOD plot for *Tnni3k* (Figure 1) shows a very sharp LOD peak with a maximum LOD score of 56.5 at rs13477506. The allele effect-size plot for the *Tnni3k* cis-eQTL at rs13477506 is shown in Figure 2A. Carriership of the 129P2-derived A-allele at the chromosome 3 locus is associated with heightened *Tnni3k* expression (Figure 2A, (Illumina probe ILMN_3023962, representative for all *Tnni3k* probe IDs) and prolonged PR interval (Figure 2B) [8]. Univariate analysis showed that the genotype at rs13477506 explains 88% (*p*<2×10^−16^) of the observed variance in *Tnni3k* transcript abundance. Differential *Tnni3k* mRNA expression was confirmed by quantitative RT-PCR in cardiac tissue (Figure 2A, dark colors).

### *In silico* haplotype analysis in a panel of inbred mice

**Figure 3:**
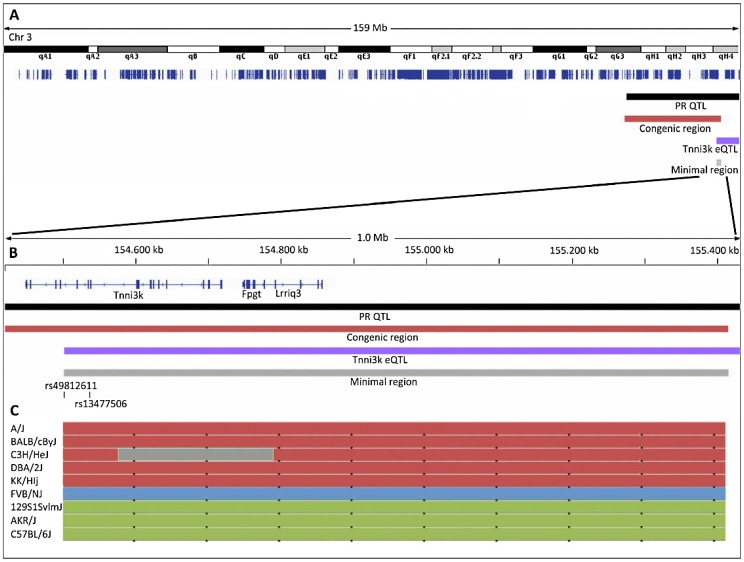
Overview of the locations of the linkage regions on chromosome 3. (A) Entire mouse chromosome 3 with refseq genes indicated in dark blue, black bar: 1.5 LOD drop of the PR-QTL; dark red bar 20 Mb DBA.AKR congenic region; purple bar 1.5 LOD drop of the Tnni3k eQTL; grey bar minimal region of overlap. (B) 1 Mb close up of the minimal region of overlap; the positions of rs49812611 (associated with nonsense mediated decay in DBA/2J) and rs13477506 (QTLs) are indicated. (C) Haplotypes of a panel of 9 inbred mouse strains as determined by the mouse phylogeny viewer (http://msub.csbio.unc.edu/) [19].

We next examined the haplotypes at the locus of interest in a panel of inbred mouse strains using the mouse phylogeny viewer [15] (Figure 3C). We identified three haplotypes in the *Tnni3K* eQTL region (indicated as red, green and blue in Figure 3C). The red haplotype (including DBA/2J) contains rs49812611, which is associated with nonsense mediated decay, leading to low levels of *Tnni3k* transcript [16]. This variant is absent in the strains with the blue and green haplotypes.

To determine whether these different genomic backgrounds in the different mouse strains were related to the PR interval we first looked at the published PR interval in publicly accessible data in the mouse phenome database [12], [17], [18]. Interestingly, this uncovered a significantly longer PR interval in inbred strains with the green (similar to 129P2) haplotype compared to strains with the red (DBA/2J) or blue (FVBN/J) haplotype (P<0.001) (www.phenome.jax.org). Since *Tnni3k* expression levels have been shown to be low in DBA/2J (red haplotype) and FVBN/J (blue haplotype) mice (which both have short PR intervals), and high in 129/P2 and AKR/J strains (both green haplotype and both having long PR interval durations) (Figure 2A and Wheeler *et al.* [16]), PR interval duration in these various mouse lines appears to correspond to the (predicted) *Tnni3k* expression levels.

### Validation of *in silico* data in inbred mice of diverse genetic background

**Figure 4:**
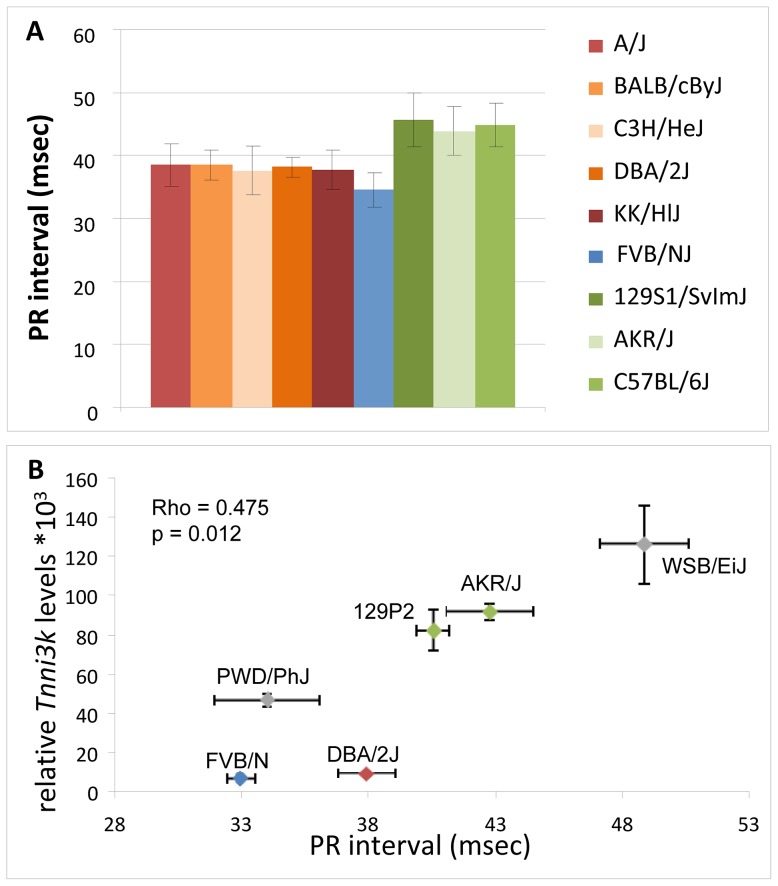
*In silico* and *in vivo* correlation of *Tnni3k* levels and PR interval duration. (A) *In silico* analysis: mouse inbred lines are colored based on the haplotypes in the minimal region of overlap; PR interval duration obtained from the mouse phenome database (http://phenome.jax.org/) FVBN/J (blue) (low expression) and green (high *Tnni3k* expression), inbred lines with the reddish colors carry rs49812611 (associated with nonsense mediated decay). (B) *In vivo* analysis of *Tnni3k* expression levels (y-axis) and PR interval duration (x-axis) in six inbred mouse lines, colors again denote the haplotype (gray is unknown haplotype).

We next validated the correlation between *Tnni3k* expression levels and PR interval duration in a diverse panel of inbred mice with the three different haplotypes (red, green and blue) and a further two wild-derived inbred lines for which no haplotype information is available (WSB/EiJ & PWD/PhJ) [19]. In these lines we determined cardiac expression levels of *Tnni3k* by qRT-PCR and measured surface ECGs for assessment of PR interval duration. As shown in Figure 4B, *Tnni3k* expression levels significantly correlate to PR interval indices (rho = 0.475, p = 0.012), thus validating the correlation observed *in silico*.

### ECG analysis in congenic DBA.AKR-*Hrtfm2* and *TNNI3K* overexpression mice

**Figure 5:**
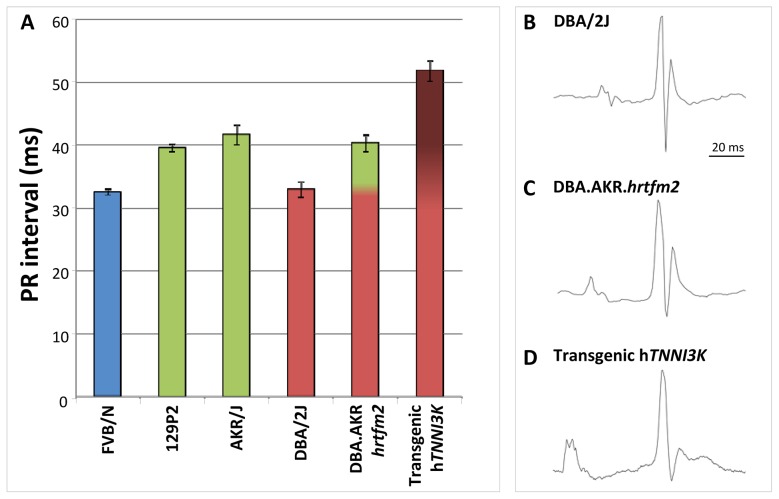
*Tnni3k* prolongs the PR interval. (A) Congenic mice carrying the AKR/J green haplotype in a DBA/2J genetic background display the green haplotype PR interval duration. Overexpression of tagged *hTNNI3k* in a DBA/2J background significantly prolongs the PR interval. Colors show the haplotype of each strain at the *Tnni3k* locus, error bars indicate standard deviations. (B–D) Examples of ECG traces of DBA/2J (B), AKR.DBA.*hrtfm2* congenic (C) and DBA/2J overexpressing h*TNNI3K* (D).

To exclude effects from loci that differ between the parental inbred strains but which map elsewhere in the genome, we measured ECGs in congenic DBA.AKR-*Hrtfm2* mice. These mice harbor approximately 20 Mb of the AKR/J (green, ‘high-*Tnni3k* expression’) haplotype at the *Tnni3k* locus in the genetic background of the DBA/2J inbred mice; pure DBA/2J mice have short PR intervals and low *Tnni3k* levels (red haplotype) (Figure 3 and Figure 4B). In DBA.AKR-*Hrtfm2* mice the level of *Tnni3k* is indistinguishable from that in AKR/J mice while the rest of the genetic background is the same as that of DBA/2J. Strikingly, DBA.AKR-*Hrtfm2* mice completely recapitulate the long PR interval of the AKR/J mice, suggesting that the difference in the level of *Tnni3k* expression is a major cause of the difference in PR interval duration between these lines (Figure 5). An overview of all the ECG paramemeters measured is given in Table 2.

**Table 2:** Overview of the ECG results mean (st.err) in DBA/2J, DBA.AKR.*hrtfm2* and transgenic *hTnni3k* mice.

Genotype	PR	QRS	QTc	HR	N
**DBA**	32.8 (0.68)	11.1 (0.31)	51.5 (1.5)	481.0 (11.6)	12
**DBA.AKR.** ***hrtfm2*** ** Congenic**	40.2 (1.2)*	12.8 (.26)	51.4 (1.6)	498.0 (14.4)	11
**Transgenic h** ***TNNI3K***	51.8 (2.4)*	19.5 (1.5)*	49.2 (2.3)	417.0 (17.0)*	8
**ANOVA**	p = <0.0001	p = <0.0001	p = 0.62	p = 0.0018	n.a.

* denotes the values significantly different from DBA/2J in the Bonferroni corrected post hoc analysis at the 0.05 significance level.

In order to exclude the contribution of any of the 117 other genes present in the congenic region to the observed effects in the DBA.AKR-*Hrtfm2* mice we measured ECGs in DBA/2J mice overexpressing human *TNNI3K.* As expected, the PR interval in these overexpression mice (with ∼20× overexpression of h*TNNI3K* [16]) was extremely prolonged (Figure 5).

### Western blot analysis of Tnni3k

**Figure 6:**
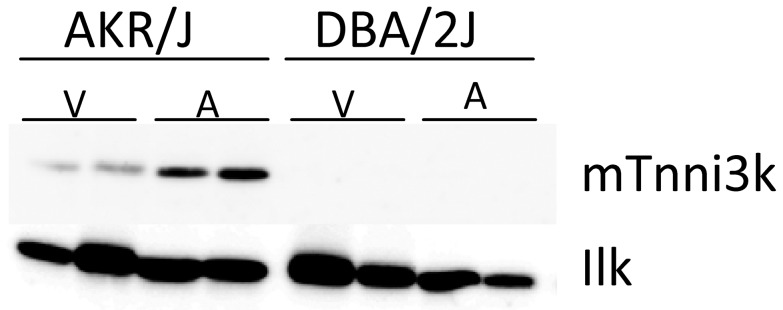
Tnni3k protein levels. Immunoblot of atrial (A) and ventricular (V) protein lysates of AKR/J and DBA/2J mice using α-mTnni3k and α-ILK as loading control.

As prolonged PR interval indicates a possible role for *Tnni3k* in atrial and/or atrio-ventricular conduction we next investigated whether Tnni3k protein is also present in the atria besides its known presence ventricle [16]. We therefore performed Western Blotting on atrial (A) and ventricular (V) protein lysates from AKR/J (high *Tnni3k* expression) and DBA/2J (low *Tnni3k* expression). Tnni3k protein was detected in both atrial and ventricular lysates in AKR/J and as expected was undetectable in DBA/2J hearts (Figure 6). In AKR/J hearts expression appeared higher in atria compared to ventricle.

## Discussion

We here dissect the PR interval QTL on mouse chromosome 3 identifying the causal gene. We used eQTL mapping to identify genes whose expression is genetically regulated by variation within the QTL region. We tested the correlation of these genes to PR interval and, based on its *in vivo* expression pattern, selected *Tnni3k* as candidate gene for the effect. We subsequently validated the role of *Tnni3k* by *in silico* haplotype-phenotype correlation and by assessing the relation between *Tnni3k* expression levels and PR interval in 6 inbred mouse lines. Finally, the causality of *Tnni3k* was proven by *in vivo* studies in (i) congenic mice harboring a “high-*Tnni3k*–long-PR-interval” haplotype in a “low-*Tnni3k*-short PR” genetic background, and (ii) mice overexpressing *Tnni3k*. These *in vivo* studies provided unequivocal evidence for the involvement of *Tnni3k* in regulation of PR interval duration.

Genetic variation is known to affect a phenotype by altering the transcriptional activity of genes [20]. Thus, eQTL mapping, integrating genome-wide transcript profiling and genetic data in a hybrid population as carried out here, provides a powerful tool for the identification of causal genes at QTLs impacting on complex traits. Moreover, coupling eQTL detection with transcript-trait correlation analysis as performed here further aids prioritization of physiologically relevant genes within the QTL region [21].

Online databases providing genotypic and phenotypic information on diverse inbred mouse lines have been instrumental in the identification and validation of the causal gene underlying the PR interval QTL. Expression pattern data deposited in the Gene Expression Omnibus (GEO) database [13], [22] aided our selection of *Tnni3k* as the prime candidate among the 4 identified eQTLs whose transcripts correlated to the PR interval. *In silico* validation of the possible influence of *Tnni3k* expression levels on the PR interval was made possible by the mouse phylogeny viewer (http://msub.csbio.unc.edu/) [19] and the mouse phenome database (http://phenome.jax.org/) [12]. Furthermore, we used the information in the phylogeny viewer to select the panel of 6 inbred mouse lines in such a way that they represented all possible independent haplotypes at the *Tnni3k* locus.

To functionally validate the role of *Tnni3k* in the *in vivo* regulation of PR interval we studied the DBA.AKR-*Hrtfm2* congenic mouse line harboring the high-*Tnni3k* expression AKR/J QTL region in the low-*Tnni3k* expression DBA/2J genetic background as well as transgenic mice overexpressing human *TNNI3K*. Strikingly, the congenic mice recapitulate perfectly the PR interval of the high-*Tnni3k* expression AKR/J donor line; furthermore, the h*TNNI3K* overexpression mice, as expected, display an extremely prolonged PR interval. Taken together this implies a dose-dependent effect of Tnni3k on atrio-ventricular conduction. Of note, h*TNNI3K* overexpression mice displayed longer QRS duration in comparison to DBA/2J wild-type mice. This concurs with our previous observation of a QTL for QRS duration post-flecainide overlapping the PR-QTL on mouse chromosome 3 [8].

*Tnni3k* was recently identified as a genetic modifier of disease progression in the *Csq^tg^* mouse model of cardiomyopathy [16]. In this model, with cardiac-specific overexpression of Calsequestrin (*Csq*), *Tnni3k* was shown to be the causative gene underlying the heart failure modifier 2 (*Hrtfm2*) locus, with high levels of *Tnni3k* accelerating the progression of the cardiomyopathic phenotype.

Little is known thus far about the physiological role of Tnni3k. *Tnni3k* encodes for cardiac Troponin I-interacting kinase 3, initially identified as a cardiac-specific protein kinase interacting with cardiac Troponin I in a yeast-two hybrid assay [23]. Its phosphorylation target(s) remain unknown [16]. The protein structure is predicted to contain an Ankyrin repeat domain besides the the Serine/Threonine-Tyrosine universal kinase domain, most likely involved in extensive protein-protein interactions.

The molecular mechanism whereby *Tnni3k* impacts on atrio-ventricular conduction requires further in-depth studies. Cardiac conduction slowing may stem from multiple mechanisms affecting cardiomyocyte depolarization, cell-cell electrical communication via gap-junctional coupling, or fibrosis, processes which may not necessarily be mutually exclusive. It is tempting to speculate that cardiac ion channels and/or gap junction proteins (connexins) may be direct targets of Tnni3k phosporylation.

In conclusion, we identified *Tnni3k* as the causal gene for a mouse PR interval QTL. Our findings provide the first evidence for *Tnni3k* in modulation of the electrocardiographic PR interval, indicating a previously unknown role for Tnni3k in atrio-ventricular conduction.

## Methods

### Mouse breeding and husbandry

The transgenic 129P2-*Scn5a* ^1798insD/+^ (129P2-MUT) and FVBN/J-*Scn5a* ^1798insD/+^ (FVBN/J-MUT) mice were generated as previously described [24]. (129P2xFVBN/J)-*Scn5a* ^1798insD/+^ F_1_ mice (F_1_-MUT) were reared from these mice, and subsequently intercrossed to produce 120 *Scn5a* ^1798insD/+^ F_2_ progeny. Generation of the congenic DBA.AKR.*hrtfm2* and h*TNNI3K*-tg mouse lines was published previously [16] The inbred strains (WSB/Eij, Molf/Eij, DBA/2j, AKR/J, PWD/PhJ) were obtained from Jackson Laboratory. All mice were supplied with the same SDS diet (SDS CRM (E) PL; Special Diets Services, UK) and water *ad libitum* and maintained on a 12-hour light/dark cycle in a temperature and humidity controlled environment. All experiments were performed in accordance with governmental and institutional guidelines for animal use in research.

### Genotyping

All F2 mice were genotyped for the *Scn5a* ^1798insD/+^ mutation as previously described [7], only mice heterozygous for the mutation were used. For the genome-wide scan, the F_2_-MUT and three mice from each parental strain were genotyped across the 19 autosomes and X chromosome by means of an Illumina Golden Gate mouse medium density (768 SNP) panel. This genotyping was carried out at Harvard Partners Center for Genetics and Genomics (HPCGG, Cambridge MA, USA). Mice with call rates <95% and single nucleotide polymorphisms (SNPs) with a call rate <95% and a minor allele frequency (MAF) <0.45 were removed from the analyses. Genotyping errors were identified using error LOD scores [13].

All analysed strains were assessed for the presence of rs49812611

### ECG measurements

ECG analysis of F_2_-MUT and inbred mice at 12 to 14 weeks of age (n = 109) was performed as previously described [10]. Briefly, mice were weighed, lightly anaesthetized by isoflurane inhalation (4.0% v/v induction; 0.8–1.2% v/v maintenance) with 800 ml/min oxygen and allowed to acclimatize for 5 minutes. The ambient temperature within the ECG recording hood was kept warm by means of a heat lamp. A 3-lead surface ECG was acquired digitally from subcutaneous 23-gauge needle electrodes at each limb of mice in the prone position using the Powerlab acquisition system (ADInstruments). Each channel was amplified and sampled at a rate of 1 kHz and a high-pass filter setting of 15 Hz. Baseline surface ECG traces were recorded for the duration of 5 minutes. A 3 minute ECG trace was analyzed for HR, and the signal average ECG (SAECG) calculated from each of leads I and II, aligned at QRS maximum, was analyzed for PR duration using the LabChart7Pro software (ADInstruments) and utilized for subsequent QTL mapping. We excluded mice that exhibited ECG parameter standard deviations greater than 1.5 ms between leads.

### Harvest and dissection of heart samples

Mice were sacrificed at 12–14 weeks by CO_2_-O_2_ asphyxiation followed by cervical dislocation. Hearts were excised, washed in 1×PBS, and dissected left ventricular (LV) free-wall flash-frozen in liquid N_2_.

### RNA preparation and microarray analysis

Total RNA was extracted from LV samples (n = 109) using the QIAGEN RNeasy mini kit 50 protocol for isolating total RNA from animal tissue using spin technology (QIAGEN Inc.) according to manufacturer's protocol. Total RNA yield (µg) and purity (260 nm∶280 nm) were determined spectrophotometrically using the NanoDrop spectrophotometer (USA). The integrity (RIN>9.0) of the re-suspended total RNA was determined using the RNA Nano Chip Kit on the Bioanalyzer 2100 and the 2100 Expert software (Agilent technologies). Synthesis, amplification and purification of anti-sense RNA was performed by using the Illumina TotalPrep RNA Amplification Kit (Ambion art. No. AM-IL1791) following the Illumina Sentrix Array Matrix expression protocol at ServiceXS (Leiden, The Netherlands). A total of 750 ng biotinylated cRNA was hybridized onto the MouseREf-8v2 Expression BeadChip (Illumina).

The raw scan data were read using the beadarray package (version 1.12.1) [14], available through Bioconductor [15]. Quality control using the function calculateBeadLevelScores showed no evidence of low-quality arrays. Illumina's default pre-processing steps were performed using beadarray. In short, estimated background was subtracted from the foreground for each bead. For replicate beads, outliers greater than 3 median absolute deviations (MADs) from the median were removed and the average signal was calculated for the remaining intensities. A variance-stabilization transformation [16] was applied to the summarized data in order to remove the mean-variance relationship in the intensities. Resulting data was then quantile normalized [17].

### eQTL mapping

eQTL mapping was performed using the R/eqtl package based on the R-statistical program, as previously described [18]. Briefly, for each transcript probe (n = 26,529) a genome-wide scan was performed with genotype and the covariates sex, weight and age as main-effects. The logarithm-of-odds (LOD) scores were calculated by interval mapping using the expectation-maximization (EM) algorithm. A multiple-transcript genome-wide significance threshold (*p*<1.88×10^−6^, Bonferroni correction for 26,529 transcript traits) was applied. Corresponding empirical LOD threshold was determined using 10,000 permutations (swapping phenotypes (ECG parameters, sex, age and weight) and genotypes, thus destroying the phenotype-genotype relationship, but maintaining the LD patterns between markers), this corresponded to a LOD score threshold of 6.83. A cis-eQTL was called when the genomic distance between the mapping SNP and transcript was less than 10 Mb [19].

### Quantitative trait transcript analysis

Transcripts for which we identified cis-eQTLs co-localizing with ECG trait QTL were tested for correlations with the respective ECG indices for conduction. Spearman's correlation coefficients (*rho*) were generated with a commercial program (SPSS software, ver. 16.0; SPSS, Chicago, IL).

### qRT–PCR

mRNA expression levels of *Tnni3k* were validated in LV tissue samples (n = 10 in each group) by quantification using the LightCycler system for real-time RT-PCR (Roche Applied Science). Quantitative PCR data was analyzed with the LinRegPCR program [2]. All samples were processed in triplicate and expression levels were normalized to GAPDH.

### Protein isolation and Western blotting

Protein was isolated from left ventricular and atrial tissue from a snap frozen mouse heart using standard procedures. In short: the tissue was homogenized in ice-cold RIPA buffer (50 mM Tris HCl pH 7.6, 150 mM NaCl, 1% NP-40, 0.5% sodium deoxycholate, 0.1% SDS) supplemented with protease inhibitors (Complete Mini; Roche) and Sodium Orthovanadate (final concentration 0.5 mM) using magnalyser ceramic beads (Roche scientific, 03358941001) for 60″. The unsoluable parts were spun down (30 sec at 4°C, 13000 rpm), the supernatant was transferred to a fresh tube and protein concentrations were determined using a BCA protein assay kit (Thermo Fisher Scientific). Protein lysates were run on a 8% acrylamide gel and blotted on a pre-equilibrated PVDF Immobilon-P membrane (Millipore) by means of a semidry system. Blots were cut at appropriate heights and probed with primary antibodies (1∶2000 α-mTnni3k rbt [16] 1∶10 000 α-ILK1 (4G9) (3856, Cell Signalling) as loading control. HRP conjugated secondary antibodies were detected with ECL-Plus (Amersham). Chemiluminescent signals were visualized using a digital image analyzer (LAS-4000 Lite; Fujifilm).

### *In silico* validation and haplotype analysis

We analyzed the haplotype structure in the 1.5 LOD drop region of the *Tnni3k* eQTL using the mouse phylogeny viewer [19]. PR interval data for 9 strains with known haplotype structure was downloaded from the mouse phenome database [12], [18] and analysed by Student's T-test (after testing for normal distribution) for differences between the strains harboring the red versus the green haplotype. FVBN/J was excluded from this test, as no data for other strains with the same haplotype was available.

### Data access

Expression data was deposited in the public gene expression omnibus (GEO) database of NCBI GEO database: GSE19741.
